# Predictors of contraceptive method discontinuation among adolescent and young women in three West African countries (Burkina Faso, Mali, and Niger)

**DOI:** 10.1186/s12905-021-01326-0

**Published:** 2021-06-29

**Authors:** Adja Mariam Ouédraogo, Adama Baguiya, Rachidatou Compaoré, Kadari Cissé, Désiré Lucien Dahourou, Anthony Somé, Halima Tougri, Seni Kouanda

**Affiliations:** 1 Institut de Recherche en Sciences de la Santé (IRSS), Ouagadougou, Burkina Faso; 2 Institut Africain de Santé Publique (IASP), Ouagadougou, Burkina Faso

**Keywords:** Contraception, Interruption, Discontinuation, Adolescent, Young women, Burkina Faso, Mali, Niger

## Abstract

**Background:**

The effective use of contraception among adolescents and young women can reduce the risk of unintended pregnancies. However, the prevalence of contraceptive use remains low in this age group. The objective of this study was to estimate the rate of contraceptive method discontinuation among adolescents and young women and to identify its associated factors in Burkina Faso, Mali, and Niger.

**Method:**

This was a secondary analysis of data from Demographic and Health Surveys of Burkina Faso (2010), Mali (2012–2013), and Niger (2012). The dependent variable was the time to discontinuation of contraceptive methods. Independent variables were represented by sociodemographic, socioeconomic, and cultural characteristics. Mixed-effects survival analysis with proportional hazards was used to identify the predictors.

**Results:**

A total of 2,264 adolescents and young women aged 15 to 24 years were included in this analysis, comprising 1,100 in Burkina Faso, 491 in Mali, and 673 in Niger. Over the last five years, the overall contraceptive discontinuation rate was 68.7% (50.1% in Burkina Faso, 59.6% in Mali, and 96.8% in Niger). At the individual level, in Burkina Faso, occupation (aHR = 0.33), number of living children (aHR = 2.17), marital status (aHR = 2.93), and region (aHR = 0.54) were associated with contraceptive discontinuation. Except for education and marital status, we found the same factors in Mali. In Niger, a women's education level (aHR = 1.47) and her partner (aHR = 0.52) were associated with discontinuation. At the community level, the region of origin was associated with discontinuation of contraceptive methods.

**Conclusion:**

Most adolescents and young women experienced at least one episode of discontinuation. Discontinuation of contraceptive methods is associated with the level of education, occupation, number of children, marital status, and desire for children with the spouse. Promotion of contraceptive interventions should target adolescents, young women, and their partners, as well as those with a low education level or in a union.

**Supplementary information:**

The online version contains supplementary material available at 10.1186/s12905-021-01326-0.

## Background

During youth (15–24 years), pregnancy can have adverse social, educational, economic, and health consequences [1–4]. The effective use of contraception among adolescents and young women can reduce the risk of unintended pregnancies [5–7]. However, the prevalence of contraception use is low in this age group. Even among those who use the methods, the discontinuation rate is high [8, 9]. Contraceptive discontinuation is defined as when an individual who "started a contraceptive method discontinued it for any reason while still at risk of getting pregnant” [10]. Contraceptive method discontinuation decreases the contraceptive prevalence and contributes to undesired fertility. It affects total fertility, unintended pregnancies, induced abortions, and miscarriages [11–15]. It decreases the effectiveness of family planning programs. Indeed, until 2010, Demographic and Health Surveys (DHS) contraceptive discontinuation estimates used to be available for only seven African countries, Egypt (2005), Ethiopia (2005), Kenya (2003), Malawi (2004), Morocco (2003), Tanzania (2004) and Zimbabwe (2005) [16]. No West African countries had these data, and these countries have the highest rates of fertility and the fastest population growth in the world [17]. Burkina Faso, Mali, and Niger are three continental countries located in West Africa. According to the *General Population and Housing Census* (*GPHC*), Burkina Faso's population was estimated at 13,730,258 inhabitants in 2006, with 14,528,662 in Mali in 2009 and 17,129,076 in Niger 2012. In these three countries, despite all family planning programs and interventions, the fertility rate remains among the highest globally, with an average number of children per woman of 6.0 in Burkina Faso, 6.1 in Mali, and 7.6 in Niger [18–22]. Despite a slight increase in the prevalence of contraceptive use, analysis of DHS data showed that the level of use of modern contraceptive methods in these three countries remains very low, at 14.3% in Burkina Faso (2010), 9.6% in Mali (2012–2013), and 11% in Niger (2012) [19–22]. Therefore, these countries have adopted several national policies, strategies, and programs related to reproductive health, population, development, and gender issues. These initiatives include the 2006–2015 Contraceptive Commodity Security Strategic Plan and 2009–2015 in Burkina Faso, the National Population Policy in Mali, and the Policy and Strategy for Repositioning Family Planning (FP) and the Government's Declaration on Population Policy in Niger [23].

Most studies on contraceptive methods uptake among adolescents and young women have not disaggregated data by age group [15, 24, 25]. In addition, the largest studies on adolescents, including the one conducted in 2009 using DHS data from 40 countries, did not consider factors associated with the discontinuation of contraceptive methods [16]. Therefore, the objective of this study was to estimate the rate of contraceptive discontinuation and identify its associated factors among adolescents and young women using DHS data from the following three West African countries: Burkina Faso, Mali, and Niger.

## Methods

### Study design and data source

This study was a secondary analysis using data from the latest DHS of three countries, including Burkina Faso (DHSBF-MICS IV, 2010), Mali (DHSM-V, 2012–2013), and Niger (DHSN-MICS IV, 2012) [19–21]. The DHS is a nationally representative population-based cross-sectional survey. On the DHS Program website, https://www.dhsprogram.com/data/, the Standard recode data files for the DHS are freely available for public access.

The procedures and reports are available and can be accessed on the DHS program website (https://dhsprogram.com/Methodology and https://dhsprogram.com/data/).

We used data collected from individual women by questionnaires on contraceptive method-related events occurring during the five years before the survey. This calendar captured monthly information on the use of contraceptive methods, the source, discontinuation, and the reason for discontinuation, as well as pregnancy, birth, and pregnancy terminations.

### Study population and sampling

**Fig. 1:**
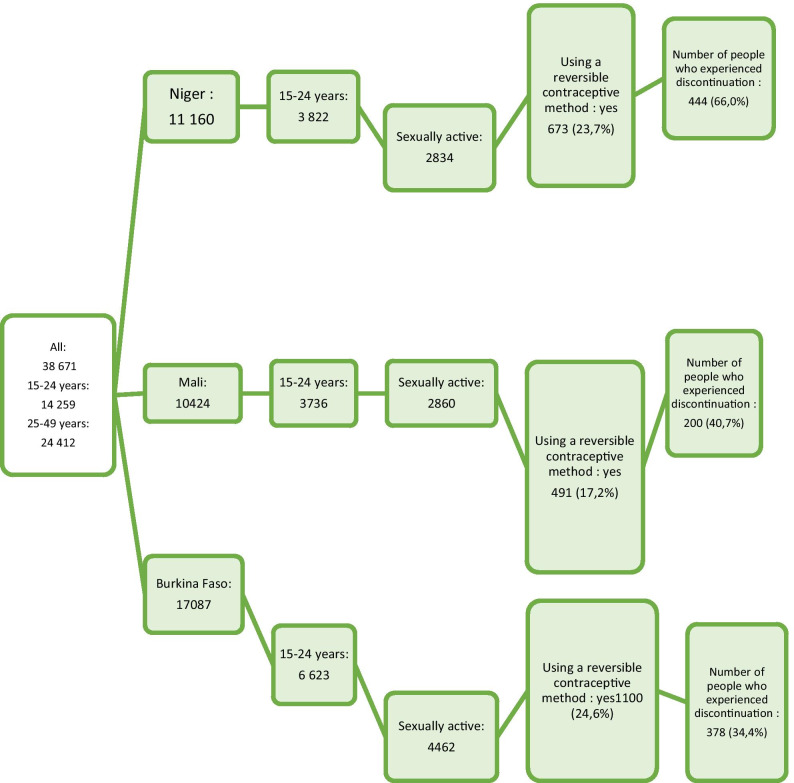
Population selection procedure

The study population was composed of all adolescents and young women in the three countries at the time of the survey. Figure 1 shows the population selection procedure. The inclusion criteria were as follows: at least 15 and no more than 24 years of age on the day of the survey; completed at least one follow-up questionnaire; sexually active and have started using a reversible contraceptive method (modern temporary female methods such as pills, intra-uterine device (IUD), injectables, implants, female condom, diaphragm, foam/jelly and Breastfeeding and Amenorrhea Method (LAM) and male condoms) during the 5-year calendar period preceding the survey.

### Study variables

In this study, the dependent variable was the time (in months) between the start of a contraceptive method and its discontinuation: the difference between the month of first use and the month of last use. We used the definition proposed by Family Planning 2020 as our dependent variable [10].

Independent or explanatory variables were divided into individual (age, education level, occupation, number of living children, marital status, access to media, desire for a spouse's child, sex of the head of household, ideal family size, health decision-making, decision-making in the household, decision-making in the family, partner's education level, and wealth quintile) and community (place of residence and region) levels.

### Data management and quality control

We selected and extracted variables related to contraceptive use discontinuation and performed additional data cleaning, labeling, and coding of all selected variables.

### Data analysis

An “episode of use” was defined as a continuous period of contraceptive use; conversely, an “episode of discontinuation” was the time between the start and end of use. Contraceptive discontinuation episodes were considered as units for analysis.

We considered data from women who began using contraception during the calendar period and stopped within three months before the interview were included. According to the literature, the three months immediately preceding the interview were excluded to avoid underestimating contraceptive failure by avoiding possible pregnancies [26].

All episodes of contraceptive method use during the five-year observation period preceding the survey (Mali: January 2007-December 2012; Niger: January 2007-December 2012 and Burkina Faso: January 2005-December 2010) were included in the calculation of the dropout rate. Thus, the unit of analysis varied from 3- to 62-month episodes of contraceptive method use.

Descriptive statistics were used to summarize the distribution of selected characteristics of women. Then, we calculated the rate of contraceptive discontinuation as the risk of discontinuation during follow-up proposed by Blanc et al. [26]. We used a competitive risk model with the "*stcompet*" command in STATA 15.1 to estimate the risk of contraceptive discontinuation.

In the univariate analysis, a mixed-effects survival model of proportional hazards was performed to explore the association between the dependent and independent variables to identify potential variables with *p*-values ≤ 0.20 to be considered in the final model.

In the multilevel analysis, we used the mixed-effects survival model of proportional hazards with the "stmixed" command in STATA 15.1.[27].

The three models were subjected to multilevel analysis.

In addition to estimating the variance of the random intercept, the models include intraclass correlation coefficients. While the intercept variance reflects heterogeneity between groups, these coefficients represent the intergroup variance ratio to the total variance and reflect the level of homogeneity within a group.

A bottom-up step-by-step procedure was used for the selection of the final model.

We assessed the model goodness of fit with Akaike Information Criteria (AIC) and tested the proportional risk assumption using “stphtest” in STATA 15.1.

We are considered the individual weight for women (v005) in the analyze.

### Ethical considerations

We sought and obtained permission from the DHS program for this secondary analysis. After approval of the proposed analysis, the program provided a link to download datasets from the three DHS programs. The primary DHS sought and obtained all relevant permissions and ethical approval before the start of the surveys.

## Results

### Sample description

A total of 2,264 adolescents and young women aged 15 to 24 were analyzed: 1,100 in Burkina Faso, 491 in Mali, and 673 in Niger. Overall, the average age was 20 ± 2.4 years.

### Individual and community characteristics

Table 1 summarizes the individual and community characteristics by country.

**Table 1:** Characteristics and factors associated with discontinuation of contraceptive methods in univariate analysis

	All				Burkina Faso				Mali				Niger			
	(n =2264)%	HR	p	[CI95%]	(n =1100)%	HR	p	[CI95%]	(n =491)%	HR	p	[CI95%]	(n =673)%	HR	p	[CI95%]
Age			0.000				0.003				0.000				0.343	
20–24 (ref. 15–19)	75.8	1.43	0.000	[1.19–1.72]	76.5	1.59	0.005	[1.15–2.20]	69.9	2.02	0.001	[1.34–3.03]	79.2	0.88	0.336	[0.67–1.15]
Education level			0.000				0.000				0.002				0.013	
Without formal education	46.8	1			40.3	1			42.6	1			60.5	1		
Primary	19.6	0.83	0.034	[0.71–0.99]	21.9	0.66	0.004	[0.51–0.87]	11.2	1.35	0.178	[0.87–2.08]	21.9	1.33	0.02	[1.05–1.70]
Secondary and higher	33.6	0.45	0.000	[0.38–0.53]	37.8	0.43	0.000	[0.33–0.56]	46.2	0.63	0.009	[0.45–0.89]	17.6	0.8	0.164	[0.58–1.10]
Occupation of the woman			0.000				0.000				0.442				0.79	
Unemployed	49.2	1			31.7	1			55	1			73.6	1		
Sales/Service	24.2	0.86	0.065	[0.74–1.01]	29.2	1.26	0.096	[0.96–1.66]	24.2	0.96	0.816	[0.66–1.39]	15.9	1.13	0.32	[0.88–1.45]
Farmer/Manual	18.4	0.73	0.001	[0.61–0.89]	32	1.29	0.071	[0.98–1.71]	5.5	1.19	0.578	[0.64–2.22]	5.9	0.98	0.923	[0.64–1.49]
Others^#^	8.2	0.5	0.000	[0.36–0.68]	7.1	0.44	0.009	[0.23–0.81]	15.3	0.69	0.161	[0.41–1.16]	4.6	1.08	0.795	[0.61–1.91]
Number of living children			0.000				0.000				0.000				0.705	
No children	25	1			38.8	1			21.8	1			4.9	1		
1 or 2	62.5	4.1	0.000	[3.31–5.09]	55.8	3.59	0.000	[2.73–4.72]	60.5	6.17	0.000	[3.11–12.26]	74.9	1.02	0.931	[0.64–1.63]
3 and more	12.5	5.16	0.000	[4.01–6.63]	5.4	4.75	0.000	[3.07–7.34]	17.7	6.27	0.000	[3.03–12.99]	20.2	1.12	0.645	[0.68–1.84]
Marital status			0.000				0.000				0.000				0.543	
In union (ref. No union)	76	4.42	0.000	[3.53–5.53]	64.6	4.56	0.000	[3.35–6.21]	74.5	3.13	0.000	[1.97–4.96]	95.7	0.85	0.533	[0.52–1.40]
Media access			0.935				0.626				0.587				0.574	
Yes (ref. No)	95.1	0.99	0.935	[0.72–1.35]	95.1	1.14	0.632	[0.67–1.92]	95.3	0.8	0.575	[0.38–1.72]	94.9	0.88	0.567	[0.56–1.38]
Desire for children of spouse			0.000				0.000				0.000				0.382	
Same for the couple	27.1	1			36	1			20.8	1			17.3	1		
Husband wants more	22.5	1.59	0.000	[1.33–1.91]	15.7	1.28	0.105	[0.95–1.71]	23	2.21	0.001	[1.40–3.48]	33.3	1.08	0.578	[0.81–1.44]
Husband wants fewer	3.4	1.52	0.009	[1.11–2.09]	3.5	1.22	0.389	[0.771.93]	4.1	2.63	0.002	[1.41–4.90]	2.8	1.54	0.212	[0.78–3.03]
Other^##^	24	0.29	0.000	[0.23–0.37]	35.5	0.24	0.000	[0.17–0.33]	25.5	0.51	0.016	[0.29–0.88]	4.3	1.12	0.682	[0.65–1.92]
Don't know	22.9	1.4	0.000	[1.18–1.67]	9.4	1.12	0.505	[0.81–1.54]	26.7	1.55	0.064	[0.97–2.45]	42.3	0.91	0.485	[0.69–1.19]
Ideal family size			0.000				0.000				0.009				0.014	
0–3	17.7	1			28.2	1			14.1	1			3.3	1		
4 or 5	45.4	1.46	0.001	[1.16–1.85]	56	1.34	0.041	[1.01–1.76]	56.2	0.98	0.953	[0.58–1.68]	20.1	2.14	0.099	[0.87–5.28]
6 and more	33.6	3.34	0.000	[2.65–4.20]	14.7	2.57	0.000	[1.84–3.60]	27.5	1.72	0.056	[0.99–3.00]	68.9	2.79	0.023	[1.15–6.77]
Divine decision	3.3	3.34	0.000	[2.27–4.93]	1.1	0.74	0.717	[0.14–3.83]	2.2	1.99	0.162	[0.76–5.20]	7.7	2.96	0.025	[1.14–7.66]
Health decision making			0.000				0.000				0.000				0.487	
Woman herself	4.4	1			3.4	1			5.5	1			5.3	1		
Woman and her partner	11	0.97	0.88	[0.69–1.38]	11.3	1.11	0.744	[0.59–2.08]	6.1	1.73	0.274	[0.65–4.60]	14.3	0.82	0.421	[0.51–1.32]
Partner only	57.7	1.08	0.627	[0.79–1.47]	47.8	1.24	0.47	[0.69–2.24]	59.7	1.62	0.223	[0.75–3.52]	72.5	0.79	0.256	[0.53–1.19]
Other person	26.8	0.29	0.000	[0.20–0.41]	37.5	0.31	0.000	[0.16–0.57]	28.7	0.53	0.141	[0.23–1.24]	7.9	1	0.995	[0.59–1.69]
Household decision making			0.000				0.000				0.000				0.826	
Woman herself	3.2	1			1.7	1			5.5	1			3.9	1		
Woman and her partner	13	0.8	0.228	[0.55–1.15]	12	0.46	0.019	[0.24–0.88]	8.8	1.47	0.414	[0.59–3.67]	17.7	1.03	0.899	[0.61–1.75]
Partner only	56.4	0.84	0.316	[0.60–1.18]	48	0.58	0.077	[0.32–1.06]	56	1.24	0.596	[0.56–2.76]	70.4	0.94	0.81	[0.58–1.53]
Other person	27.4	0.23	0.000	[0.16–0.33]	38.3	0.14	0.000	[0.07–0.26]	29.7	0.47	0.085	[0.20–1.11]	8	1.09	0.784	[0.60–1.97]
Family visiting decisions			0.000				0.000				0.000				0.439	
Woman herself	16.5	1			23.3	1			8.6	1			11.3	1		
Woman and her partner	15.9	1.39	0.002	[1.13–1.72]	11.1	1.07	0.709	[0.75–1.51]	10.4	1.2	0.616	[0.602.40]	27.8	0.98	0.905	[0.70–1.37]
Partner only	41.2	1.28	0.007	[1.07–1.52]	28.1	1.11	0.443	[0.85–1.44]	53	1.18	0.565	[0.67–2.07]	54.1	0.83	0.24	[0.61–1.13]
Other person	26.3	0.3	0.000	[0.24–0.39]	37.4	0.24	0.000	[0.17–0.33]	28.1	0.41	0.008	[0.21–0.80]	6.8	0.89	0.643	[0.55–1.44]
Partner education level			0.000				0.000				0.000				0.025	
Uneducated	41.4		1		32	1			40.5	1			57.5	1		
Primary	15	0.82	0.034	[0.69–0.99]	13.7	0.7	0.03	[0.51–0.97]	9.8	1.01	0.972	[0.63–1.62]	20.9	0.9	0.421	[0.70–1.16]
Secondary and higher	19.4	0.65	0.000	[0.54–0.77]	18.6	0.72	0.019	[0.54–0.95]	24.2	0.79	0.261	[0.53–1.19]	17.3	0.63	0.004	[0.46–0.86]
No partner	24.2	0.19	0.000	[0.15–0.24]	35.7	0.18	0.000	[0.13–0.25]	25.5	0.3	0.000	[0.19–0.49]	4.4	1.05	0.848	[0.63–1.74]
Wealth quintile			0.000				0.000				0.088				0.567	
Poorer	6.9	1			5.4	1			6.7	1			9.7	1		
Poor	9.2	0.92	0.554	[0.69–1.22]	8.7	0.76	0.285	[0.45–1.26]	9.4	0.99	0.978	[0.52–1.89]	9.8	1.06	0.787	[0.71–1.57]
Medium	11.0	0.78	0.079	[0.60–1.03]	10.5	0.65	0.082	[0.40–1.06]	9.8	0.58	0.128	[0.29–1.17]	12.8	1.05	0.788	[0.73–1.53]
Rich	21.7	0.73	0.01	[0.57–0.93]	20.8	0.55	0.007	[0.35–0.85]	23.8	0.66	0.17	[0.37–1.19]	21.5	1.25	0.2	[0.89–1.76]
Richer	51.2	0.48	0.000	[0.38–0.60]	54.6	0.35	0.000	[0.23–0.52]	50.3	0.55	0.037	[0.31–0.97]	46.2	1.01	0.961	[0.73–1.40]
Sex of head of household			0.229				0.010				0.709				0.411	
Female (ref. Male)	11.9	0.89	0.235	[0.73–1.08]	13	0.64	0.012	[0.45–0.91]	11	0.9	0.712	[0.53–1.55]	10.7	1.12	0.404	[0.86–1.47]
Place of residence			0.000				0.000				0.431				0.702	
Rural (ref. Urban)	46.4	1.65	0.000	[1.44–1.89]	39.8	1.69	0.000	[1.36–2.11]	45	1.13	0.432	[0.83–1.55]	58.1	1.04	0.702	[0.84–1.30]
Region^###^							0.011				0.17				0.002	
0					18.6	1			37.5	1			19.3	1		
1					5	2.64	0.000	[1.72–4.05]	12.2	1.01	0.988	[0.57–1.77]	6.1	0.22	0.011	[0.07–0.70]
2					9.6	0.83	0.526	[0.48–1.46]	13.4	0.65	0.092	[0.39–1.07]	6.4	0.6	0.205	[0.28–1.31]
3					2.6	1.71	0.143	[0.83–3.52]	13	1.2	0.429	[0.77–1.87]	16.2	1.15	0.44	[0.81–1.62]
4					2.3	1.71	0.173	[0.79–3.68]	15.1	0.92	0.719	[0.57–1.48]	14.9	0.8	0.199	[0.56–1.12]
5					9.7	1.26	0.294	[0.82–1.92]	8.8	1.44	0.212	[0.81–2.56]	11.3	1.34	0.109	[0.94–1.93]
6					7.7	1.17	0.582	[0.67–2.05]					8.3	1.03	0.878	[0.67–1.59]
7					6	1.24	0.341	[0.79–1.94]					17.5	1.04	0.802	[0.76–1.43]
8					16.8	0.92	0.608	[0.67–1.27]								
9					5.5	0.76	0.423	[0.39–1.49]								
10					5.5	1.13	0.701	[0.60–2.14]								
11					3.3	1.53	0.197	[0.80–2.92]								
12					7.4	0.88	0. 708	[0.44–1.74]								
Countries			0.000													
Burkina Faso		1														
Mali		1.61	0.000	[1.33–1.94]												
Niger		2.9	0.000	[2.50–3.37]												

Other# manager/technician, jobs others, Other##: no partner, not envisaged

Region^###^: Burkina Faso: 0 = Centre 1 = Boucle du Mouhoun, 2 = Cascades, 3 = Centre-Est, 4 = Centre-Nord, 5 = Centre-Ouest, 6 = Centre-Sud, 7 = Est, 8 = Hauts Bassins, 9 = Nord, 10 = Plateau Central, 11 = Sahel et 12 = Sud-Ouest

Mali: 0 = Bamako, 1 = Kayes, 2 = Koulikoro, 3 = Sikasso, 4 = Ségou, 5 = Mopti

Niger: 0 = Niamey, 1 = Agadez, 2 = Diffa, 3 = Dosso, 4 = Maradi, 5 = Tahoua, 6 = Tillabéry, 7 = Zinder

Overall, 75.8% of the users were aged 20 to 24. In Niger, most adolescents and young women had no formal education. Unemployed female users were the most represented, with 31.7% in Burkina Faso, 55.0% in Mali, and 73.6% in Niger.

### Contraceptive discontinuation rate

In the last five years, the discontinuation rate was 50.1% in Burkina Faso, 59.6% in Mali, and 96.8% in Niger (Table 2). The desire to become pregnant (Burkina Faso: 20.2%; Mali: 22.1% and Niger: 44.4%) was the main reason for discontinuation, followed by the side effects of the methods (8.7% for Burkina Faso and 10.5% for Mali).

**Table 2:** Contraceptive discontinuation rate per 100 episodes by reason cited according to country and overall

	All (%)	Burkina Faso (%)	Mali (%)	NIGER (%)
Failure of the method	3.1	3.3	5.9	1.7
Changed method ^####^	5.4	3.9	3.8	8.4
Wanted to get pregnant	29.6	20.2	22.1	44.4
Other reasons related to fertility^#####^	7.1	4.7	3.1	12.9
Side effects/health problem	6.8	8.7	10.5	2.5
Need for a more effective method	3.5	1.9	3.4	5.7
Other reasons related to the method^######^	2.6	2.7	3.1	2.2
Other reasons	15.9	8.6	11.4	27.5
All reasons	68.7	50.1	59.6	96.8

^####^ Used a different method within one month of discontinuation or said she wanted a more effective method and started using another method within two months of interruption/#####Includes infrequent sex/husband absent, difficulty getting pregnant/and dissolution of union/separation, ######Includes lack of access/remote, too expensive and inconvenient use

### Factors associated with contraceptive discontinuation

In the univariate analysis (Table 1), the factors associated with contraceptive discontinuation were as follows:

- Individual factors: age (HR = 1.43; 95% CI [1.19–1.72]; *p* < 0.001 for 20–24 old), education level of the female (HR = 0.45; 95% CI[0.38–0.53]; *p* < 0.001 for secondary and higher), type of occupation (HR = 0.73; 95% CI[0.61–0.89]; *p* < 0.001 for farmer/manual), number of living children (HR = 5.16; 95% CI [4.01–6.63]; *p* < 0.001 for), marital status (HR = 4.42; 95% CI [3.53–5.53]; *p* < 0.001 for in union), and wealth quintile (HR = 0.48; 95% CI [0.38–0.60]; *p* < 0.011 for richer);
- Factors related to their partner: partner's desire for children (HR = 1.59; 95% CI [1.33–1.91]; *p* < 0.001 for situation of husband wants more children), partner's level of education (HR = 0.65; 95% CI [0.54–0.77]; *p* < 0.011 for secondary and higher level), and ideal family size as reported by the woman (HR = 3.34; 95% CI [2.65–4.20]; *p* < 0.001 for 6 and more children).
- Linked to decision-making power in the couple: decision-making regarding health care (HR = 0.29; 95% CI [0.20–0.41; *p* < 0.001 for other person), decision-making in the household (HR = 0.23; 95% CI [0.16–0.33]; *p* < 0.001 for other person), and decision-making for family visits (HR = 1.28; 95% CI [1.07–1.52]; *p* < 0.01 for partner only);
- Residence: place of residence (HR = 1.65; 95% CI [1.44–1.89]; *p* < 0.001 for rural) and country (HR = 2.9; 95% CI [2.50–3.37]; *p* < 0.001 for Niger).

Table 3 shows the adjusted hazard ratios by country as well as for the pooled analysis. The pooled data show that the factors associated with discontinuation of contraceptive methods in the adjusted model were the level of education (aHR = 1.21; 95%, *p* < 0.05 for those with primary education), woman's occupation (aHR = 0.69; *p* < 0.01 for technicians and others), number of living children (aHR = 1.71; *p* < 0.01 for those with three or more children), marital status (aHR = 2.21;; *p* < 0.01 for women in a union), desire for children of the spouse (aHR = 1.14; *p* < 0.001 for those whose their partner wanted more), partner's level of education (aHR = 0.80; *p* < 0.05 for high school and above) and country of origin (Table 3).

**Table 3:** Adjusted hazard ratios of the final multilevel multivariate model identifying factors associated with contraceptive discontinuation

	Ensemble	Burkina Faso	Mali	Niger
	aHR	aHR	aHR	aHR
*Age*				
20-24 (ref. 15-19)	0.89	0.95		0.78
*Education level*				
Without formal education	1	1	1	1
Primary	1.21*	0.88	1.57	1.47***
Secondary and higher	1.1	1.04	1.51	1.09
*Occupation of the woman*				
Unemployed	1	1		
Sales/Service	1.01	0.96		
Farmer/Manual	0.96	0.93		
Other^#^	0.69**	0.33**		
*Number of living children*				
No children	1	1	1	1
01 or 02	1.60***	1.51*	2.34**	0.9
3 and more	1.71**	2.17**	2.35*	1.02
*Marital status*				
In union (ref. No union)	2.21**	2.92***	1.04	
*Desire for spouse's children*				
Same for the couple	1	1	1	
Husband wants more	1.14***	1.50**	1.95*	
Husband wants fewer	1.2	1.29	1.52	
others	Omis	Omis	Omis	
Don't know	1.09	1.04	1.49	
*Ideal family size*				
0-3	1		1	1
4 or 5	0.85		0.71	1.29
Divine decision				
*Household decision making*				
Woman herself				
Woman and her partner				
Partner only				1.31
Other person				1.31
*Partner education level*				
Uneducated	1	1	1	1
Primary	0.91	0.87	0.86	0.87
Secondary and higher	0.80*	1.08	0.72	0.52***
No partner	Omis	Omis	Omis	1.37
*Wealth quintile*				
Poorer	1	1	1	
Poor	0.93	1.17	0.79	
Medium	0.86	0.97	0.63	
Rich	0.88	0.97	0.76	
Richer	0.78	0.58	0.72	
*Sex of head of household*				
Female (ref. Male)	1.01	1.11		0.85
*Place of residence*				
Rural (ref. Urban)	0.98	0.82	1.15	0.92
*Region#*				
0		1	1	1
1		1.65	0.69	0.24***
2		0.66	0.48*	0.51*
3		1.11	0.79	1.03
4		0.88	1.01	0.73
5		0.91	0.93	1.33
6		0.99		1.16
7		0.54*		0.96
8		0.8		
9		0.47*		
10		0.64		
11		0.69		
12		0.89		
*Countries*				
Burkina Faso	1			
Mali	1.56***			
Niger	1.51***			
*Random effects*				
Variance of the intercept (standard deviation	0.04 (0.03) ***	0.07 (0.06) ***	0.32 (0.14) ***	0.02 (0.04)***
ICC (%)	1.2	2.08	8.86	0.6

Other# manager/technician. jobs others

According to the respondent's statement. * p < 0.05. **p ≤ 0.01. *** p < .001

ref. = reference group

na = not applicable

aHR: Adjusted hazard ratio

According to the respondent's statement. * p < 0.05. **p ≤ 0.01. *** p < .001. ref. = reference group. na = not applicable HRaj: Adjusted hazard ratio

## Discussion

Our results show that over the past five years preceding the survey, more than half of the adolescents and young women had experienced at least one discontinuation of contraception. The predictors of discontinuation included both individual and community level variables in all three countries.

The individual factors associated with discontinuation were primarily the adolescents’ and young women's occupation, marital status, partner's desire for a child, and education. These factors differed slightly across the three countries. They were all observed in Mali. Education level was associated with discontinuation in Niger, but not in Burkina Faso.

At the community level, the region was associated with the discontinuation of contraceptive methods. Compared to those living in capital cities, those living in other regions, such as the eastern and northern regions of Burkina Faso, Koulikoro region of Mali Agadez, and Diffa regions of Niger, were less likely to discontinue their contraceptive methods.

Our results show that more than one-third of the study participants surveyed had experienced at least one contraceptive discontinuation in each of the three countries. This result could be explained by the failure to consider the specific needs of adolescents and young women for family planning interventions, particularly regarding the adequacy of available methods with the needs or preferences of young people. In contrast, there could be challenges with the quality of the services offered to them. In our context, clients do not get enough counseling after the start of a contraceptive method [28]. Meanwhile, previous studies reported that participants who received more information and better counseling about side effects are less likely to stop using contraception [29]. Adolescents and young women need better information about contraception, whether provided as part of sexual and reproductive health education in schools, through the media, or in health facilities. This should address common concerns about the side effects of contraception and how these can be managed without compromising effectiveness.

Surprisingly, a high level of education was associated with an increased likelihood of discontinuation of contraceptive methods. One might think that those who are more educated would have more opportunities to interact with health workers more effectively and access various sources of information about modern contraception. This is expected to lead to a choice of and adherence to a suitable contraceptive method. However, it appeared that women who were more educated and aware of the side effects of contraceptive methods may opt to discontinue their use and choose a method that offers less potential harm.

Nonetheless, our results are similar to those of previous studies conducted in sub-Saharan Africa. Curtis et al. found in a multi-country analysis of the DHS data that educated women of childbearing age in Egypt and Zimbabwe were more likely to discontinue contraceptive methods [30]. Similarly, in an analysis of DHS data from Senegal and Ethiopia, Barden-O'Fallon et al. and Alvergne et al. found that women of reproductive age with higher education levels were approximately 50% more likely to discontinue contraceptive methods [31, 32]. In contrast, working Adolescents and young women were less likely to discontinue contraceptive methods, as reported in the literature [24].

Our study also suggests that girls from the wealthiest households were less likely to discontinue their contraceptive method. This is in agreement with two previous studies conducted in eight countries and Ethiopia [24, 32]. Economic activity not only offers freedom but also the means to pay for contraceptive methods. The fact that modern contraceptives are not free of charge can be an obstacle to their use, especially for adolescents and young women who are still in school or jobless[33]. However, unlike our results, some authors found no association between household standard of living and the discontinuation of contraceptive methods among women of reproductive age [31, 34, 35].

Adolescents and young women in a union were more likely to discontinue their contraceptive methods. This finding is supported by other studies [13, 31, 36]. In addition, women who report their partners' desire for more children were more likely to discontinue their contraceptive methods than those who perceive their partners would want the same number of children. Similar studies from different settings have also supported these findings [34, 37]. This implies that males are predominant decision-makers of contraception use in these countries. However, Ali et al. showed that among women of childbearing age, a partner's desire to have more children than his or her spouse is associated with the discontinuation of contraceptive methods in some countries (Kenya, Bangladesh, and Indonesia) [14, 24].

Moreover, adolescents and young women whose partner/spouse had secondary school or higher education were less likely to discontinue contraceptive methods. The same result was found by Alem et al. in Ethiopia [36]. In contrast, an analysis of Bangladesh DHS data by Khan et al. found that women whose husbands had secondary school education or higher were more likely to discontinue their contraceptive methods [38]. These different findings imply that continuity of contraceptive use among adolescents and young women remains conditioned by contextual and sociocultural factors. Indeed, fertility is still highly valued in many developing countries, and the influence and decision-making power of the partner remain factors to be considered.

Our study highlights the importance of evaluating various factors when developing youth reproductive health programs by considering their specific needs and improving the quality of the services, including financial accessibility, communication, and the partners’ involvement. Other sectoral actions, such as female empowerment and education level improvements, should also be developed.

The present study has some limitations. First, women may not want to report their experience of contraception discontinuation due to social reasons. Second, the potential for recall bias was minimized by including only the use of the most recent contraceptive methods, and data were collected monthly. However, the findings offer an opportunity to provide evidence-based data needed to improve the preparation and implementation of family planning programs targeting adolescents and young women.

## Conclusion

Contraceptive method discontinuation among adolescents and young women in Burkina Faso, Mali, and Niger is high. The factors associated with discontinuation, at the individual level, include the girl's occupation, marital status, and partner's desire for a child in Burkina Faso. In Mali, these same factors were observed. In Niger, the associated factors included the levels of education of the adolescents and young woman and her partner. At the community level, the region was associated with discontinuation of contraceptive methods. To achieve Sustainable Development Goals, particularly improvement of continued use of contraceptive methods, factors that increase the discontinuation rate should be considered in family planning promotion programs.

## Supplementary information

**Additional file 1:** Sampling and sampling of clusters, households and women of childbearing age in the three countries information.

## Data Availability

The dataset supporting the conclusions of this article is freely available to the public at https://dhsprogram.com and can be accessed after a request is made to and approved by the Demographic and Health Surveys program.

## Abbreviations

AIC: Akaike information criteria
CI: Confidence interval
DHS: Demographic and Health Survey
GPHC: General Population and Housing Census
HR: Hazard Ratio
aHR: Adjusted hazard ratio
IASP: Institut Africain de santé publique
ICC: Intraclass correlation coefficient
IRSS: Institut de Recherche en sciences de la santé
PVV: Proportional variance variation

## References

[1] Blanc AK. Excess risk of maternal mortality in adolescent mothers. Lancet Glob Health [Internet] 2014 [cited 2016 May 24];2(4):e201. http://www.thelancet.com/journals/langlo/article/PIIS2214-109X(14)70028-2/abstract 10.1016/S2214-109X(14)70028-2 25103056

[2] Nove A, Matthews Z, Neal S, Camacho AV. Maternal mortality in adolescents compared with women of other ages: evidence from 144 countries. Lancet Glob Health [Internet] 2014 [cited 2016 Oct 17];2(3):e155–64. http://www.sciencedirect.com/science/article/pii/S2214109X13701797 10.1016/S2214-109X(13)70179-7 25102848

[3] Urdinola BP, Ospino C (2015). Long-term consequences of adolescent fertility: the Colombian case. Demogr Res.

[4] Gibb SJ, Fergusson DM, Horwood LJ, Boden JM (2015). Early motherhood and long-term economic outcomes: findings from a 30-year longitudinal study. J Res Adolesc.

[5] UNFPA. The power of 1.8 Billion : Adolescents,youth and the transformation of the future. 2014.

[6] Damle LF, Gohari AC, McEvoy AK, Desale SY, Gomez-Lobo V (2015). Early initiation of postpartum contraception: does it decrease rapid repeat pregnancy in adolescents?. J Pediatr Adolesc Gynecol.

[7] Winner B, Peipert JF, Zhao Q, Buckel C, Madden T, Allsworth JE (2012). Effectiveness of long-acting reversible contraception. N Engl J Med.

[8] Wilson EK, Samandari G, Koo HP, Tucker C (2011). Adolescent mothers’ postpartum contraceptive use: a qualitative study. Perspect Sex Reprod Health.

[9] Maslyanskaya S, Coupey SM, Chhabra R, Khan UI (2016). Predictors of early discontinuation of effective contraception by teens at high risk of pregnancy. J Pediatr Adolesc Gynecol.

[10] Family Planning 2020. Contraceptive discontinuation: Reasons, challenges, and solutions. 2015; http://www.familyplanning2020.org/resources/12019

[11] Cleland J, Ali MM (2004). Reproductive consequences of contraceptive failure in 19 developing countries. Obstet Gynecol.

[12] Blanc AK, Curtis SL, Croft T. Does contraceptive discontinuation matter?: Quality of care and fertility consequences. MEASURE Evaluation, Carolina Population Center, University of North Carolina at Chapel Hill Chapel Hill, NC, USA; 1999.

[13] Curtis S, Evens E, Sambisa W. Contraceptive Discontinuation and Unintended Pregnancy: An Imperfect Relationship. [cited 2017 Sep 15]; http://citeseerx.ist.psu.edu/viewdoc/download?doi=10.1.1.591.943&rep=rep1&type=pdf 10.1363/3705811 PMC3912075 21757420

[14] Ali MM, Cleland JG, Shah IH. Causes and consequences of contraceptive discontinuation: evidence from 60 demographic and health surveys. WORLD Health Organ [Internet] 2012 [cited 2015 Aug 14];197. http://apps.who.int/iris/handle/10665/75429

[15] Jain AK, Winfrey W. Contribution of contraceptive discontinuation to unintended births in 36 developing countries. stud fam plann [Internet] 2017;48(3):269–78 10.1111/sifp.12023 28398595

[16] Blanc AK, Tsui AO, Croft TN, Trevitt JL. Patterns and trends in adolescents’ contraceptive use and discontinuation in developing countries and comparisons with adult women. Int Perspect Sex Reprod Health [Internet] 2009 [cited 2015 Aug 21];63–71. Available from: http://www.jstor.org/stable/40233806 10.1363/ipsrh.35.063.09 19620090

[17] Aisha D, Michelle W, Ben B, Win B. New users” are confusing our counting: reaching consensus on how to measure “additional users” of family planning. Glob. Health Sci. Pract.2017;6–14. 10.9745/GHSP-D-16-00328 PMC5478230 28351876

[18] Darroch JE, Singh S (2013). Trends in contraceptive need and use in developing countries in 2003, 2008, and 2012: an analysis of national surveys. The Lancet.

[19] Cellule de Planification et de Statistique (CPS/SSDSPF), Institut National de la Statistique (INSTAT/MPATP), INFO-STAT, ICF International. Enquête Démographique et de Santé au Mali 2012–2013. Rockville, Maryland, USA: CPS, INSTAT, INFO-STAT et ICF International; 2014.

[20] INDS et MACRO. Enquête démographiques et de santé et à indicateurs multiples IV Burkina Faso 2010. Ouagadougou: 2012.

[21] Institut National de la Statistique (2013). ICF International, Enquête Démographique et de Santé et à Indicateurs Multiples (EDSN-MICS IV) 2012, Niger.

[22] INSD. Recensement générale de la population et de l’Habitation 2006. 2008;52.

[23] INDS et MACRO. Enquête démographiques et de santé et à indicateurs multiples IV Burkina Faso 2010.

[24] Bradley SE, Schwandt H, Khan S. Levels, trends, and reasons for contraceptive discontinuation. DHS Anal Stud [Internet] 2009 [cited 2017 Sep 17];20. Available from: http://cedar.wwu.edu/fairhaven_facpubs/1/

[25] Ali M, Cleland J (1999). Determinants of contraceptive discontinuation in six developing. J Biosoc Sci.

[26] K Blanc A, Curtis S, Croft T. Monitoring contraceptive continuation: links to fertility outcomes and quality of care. 2002. 10.1111/j.1728-4465.2002.00127.x 12132634

[27] Crowther MJ. Multilevel mixed effects survival analysis: Estimation, simulation and application. 2018;17.

[28] Senderowicz L (2015). Side-effects and contraceptive uptake in urban Burkina Faso. Contraception.

[29] Blackstone SR, Nwaozuru U, Iwelunmor J. Factors influencing contraceptive use in Sub-Saharan Africa: a systematic review. Int Q Commun Health Educ [Internet] [cited 2017 Feb 8];0272684X16685254. Available from: http://journals.sagepub.com/doi/abs/10.1177/0272684X16685254 10.1177/0272684X16685254 28056643

[30] Curtis SL, Blanc AK. Determinants of contraceptive failure, switching, and discontinuation: an analysis of DHS contraceptive histories. DHS Anal Rep No6 1997;

[31] Barden-O’Fallon J, Speizer IS, Calhoun LM, Corroon M. Women’s contraceptive discontinuation and switching behavior in urban Senegal, 2010–2015. BMC Womens Health [Internet] 2018 [cited 2018 Mar 10];18(1). Available from: https://bmcwomenshealth.biomedcentral.com/articles/. 10.1186/s12905-018-0529-9 10.1186/s12905-018-0529-9 PMC5800088 29402320

[32] Alvergne A, Stevens R, Gurmu E. Side effects and the need for secrecy: characterising discontinuation of modern contraception and its causes in Ethiopia using mixed methods. bioRxiv 2017;140004. 10.1186/s40834-017-0052-7 PMC5683325 29201429

[33] Susheela AEBAM, Woog SV. Adolescents’ views of and preferences for sexual and reproductive health services in Burkina Faso, Ghana, Malawi and Uganda. Afr J Reprod Health [Internet] 2007 [cited 2016 Oct 17];11(3). Available from: http://www.ajrh.info/vol11_no3/biddlecom.pdf PMC2367115 18458737

[34] Modey EJ, Aryeetey R, Adanu R (2014). Contraceptive discontinuation and switching among Ghanaian women: evidence from the Ghana Demographic and Health Survey, 2008. Afr J Reprod Health.

[35] Ali M, Cleland J. Contraceptive Discontinuation in Six Developing Countries: A Cause-Specific Analysis. Int Fam Plan Perspect [Internet] 1995 [cited 2018 Jul 2];21(3):92. Available from: https://www.jstor.org/stable/2133181?origin=crossref

[36] Alem Gebremariam TB. Factors Associated with Contraceptive Discontinuation in Agarfa District, Bale Zone, South East Ethiopia. Epidemiol Open Access [Internet] 2015 [cited 2017 Sep 17];05(01). Available from: https://www.omicsonline.org/open-access/factors-associated-with-contraceptive-discontinuation-in-agarfa-district-bale-zone-south-east-ethiopia-2161-1165.1000179.php?aid=40628

[37] Alem Gebremariam TB. Factors Associated with Contraceptive Discontinuation in Agarfa District, Bale Zone, South East Ethiopia. Epidemiol Open Access [Internet] 2015 [cited 2018 Jul 2];05(01). Available from: https://www.omicsonline.org/open-access/factors-associated-with-contraceptive-discontinuation-in-agarfa-district-bale-zone-south-east-ethiopia-2161-1165.1000179.php?aid=40628

[38] Khan MA (2003). Factors associated with oral contraceptive discontinuation in rural Bangladesh. Health Policy Plan.

