# Supplementary material for: Predictors of contraceptive method discontinuation among adolescent and young women in three West African countries (Burkina Faso, Mali, and Niger)
Source: BMC Womens Health. 2021 Jun 29;21:261. doi: 10.1186/s12905-021-01326-0 (PMC8240211; doi:10.1186/s12905-021-01326-0)
Supplement: Supplementary file 1 — Additional file 1: Sampling and sampling of clusters, households and women of childbearing age in the three countries information. [file 12905_2021_1326_MOESM1_ESM.docx]

**Predictors of contraceptive methods discontinuation among adolescent and young women in three West African countries (Burkina Faso, Mali, Niger)**

Adja Mariam OUEDRAOGO^1,2*^, Adama BAGUIYA^1,2^, Rachidatou COMPAORE^1,2^, Kadari CISSE^1^, Désire Lucien DAHOUROU^1^, Anthony SOME^1^, Halima TOUGRI^1,2^, Seni KOUANDA^1,2^

^Authors affiliation^

^1^ Institut de Recherche en Sciences de la Santé (IRSS)

^2^ Institut Africain de Santé Publique (IASP)

### * Corresponding author

Adja Mariam OUEDRAOGO : email : [adjamariam@yahoo.fr](mailto:adjamariam@yahoo.fr) TEL : +22675509960

Authors’ contact details

Adja Mariam OUEDRAOGO : email : [adjamariam@yahoo.fr](mailto:adjamariam@yahoo.fr) TEL : +22675509960

Adama BAGUIYA; email: [abaguiya@gmail.com](mailto:abaguiya@gmail.com), Tel 70878370

Rachidatou COMPAORE : email : [rachidoc7@yahoo.fr](mailto:rachidoc7@yahoo.fr) TEL : +226 72 37 56 12

Kadari CISSE: email: cisskad4@gmail.com, Tel +226 76425486

Désiré Lucien DAHOUROU: email : [ddahourou@gmail.fr](mailto:ddahourou@gmail.fr) , Tel +226 70 13 01 96

Anthony SOME: email: [sthony2000@yahoo.fr](mailto:sthony2000@yahoo.fr) , Tel +226 70695011

Halima TOUGRI: email: [tougrih@yahoo.com](mailto:tougrih@yahoo.com) , Tel +226 70240133

Séni KOUANDA: email: senikouanda@gmail.com, Tel +226 70261462

**Supplementary material**

In Burkina Faso, there are 13 administrative regions; each region is made up of one or more provinces; there are 45 provinces; each province is made up of communes, and each commune is made up of villages or sectors; the 13 health regions are: 0= Centre 1= Boucle du Mouhoun, 2=Cascades, 3= Centre-East, 4= Centre-North, 5= Centre-West, 6= Centre-South, 7= East, 8= Hauts Bassins, 9= North, 10= Central Plateau, 11= Sahel and 12= South-West;

The 2010 EDSBF-MICS IV covered the population of individuals residing in ordinary households throughout the country. The sample is stratified to provide an adequate representation of urban and rural areas as well as the 13 areas of study (consisting of the 13 administrative regions) for which estimates are available for all key indicators. It is a stratified, two-stage, areal sample.

In Mali, the survey took place in the 8 administrative regions (Kayes, Koulikoro, Sikasso, Ségou, Mopti, Timbuktu, Gao and Kidal) and the district of Bamako. Each region is made up of circles, each circle is made up of arrondissements, and each arrondissement is made up of communes. In total, there are 49 circles, 158 arrondissements and 730 communes, of which the city of Bamako is subdivided into 6 communes. The most densely populated region is the Sikasso region, which represents 18% of the total population. The least populated region is the Kidal region which represents only 0.5% of the total population. The capital Bamako represents 12.5% of the total population. In Mali, 22.6% of the population lives in urban areas, more than half of which is in Bamako.

The EDSM V 2012-2013 sample is a representative sample at the level of all five regions and the district of Bamako, based on a two-stage stratified cluster survey. As in all DHS, the EDSM V sample is also representative at the level of each area of study. This is in contrast to previous DHS which had eleven areas of study composed of each of the eight regions (Kayes, Koulikoro, Sikasso, Segou, Mopti, Gao, Timbuktu, Kidal), Bamako District, urban and rural Mali, EDSM V comprises eight fields of study made up of each of the five southern regions (Kayes, Koulikoro, Sikasso, Ségou, Mopti), the district of Bamako, and their urban and rural areas as a whole. The northern regions were not included in EDSM V.

At the administrative level, Niger has put in place a decentralisation process to create the conditions for better sharing and more organised implementation of administrative reform, to help democracy take root and to empower grassroots communities in the management of their development. Thus, the country is subdivided into 8 administrative regions. The regions are subdivided into departments (63 in number). There are 266 communes, 52 of which are urban and 214 rural.

The sample for the 2012 EDSN-MICS IV is a nationally representative sample of households, based on a two-stage stratified cluster survey. As with all past DHSs, the EDSN-MICS IV includes 10 areas of study from each of the country's eight regions (Agadez, Diffa, Dosso, Maradi, Tahoua, Tillabéri, Zinder and Niamey), urban and rural areas ^[20–22]^.

Table 1 : *Sampling and sampling of clusters, households and women of childbearing age in the three countries*

|  | Burkina Faso 2010 | Mali 2012 | Niger 2012 |
| --- | --- | --- | --- |
| Number of bunches selected in the 1st degree | 574 | 585 | 480 |
| Number of households selected | 14947 | 10743 | 11900 |
| Number of households identified | 14536 | 10265 | 10969 |
| Number of households surveyed | 14424 | 10105 | 10750 |
| Number of eligible women | 17363 | 10875 | 11698 |
| Number of women surveyed | 17087 | 10424 | 11160 |
| Rate of responses from eligible women surveyed (%) | 98,4 | 95,9 | 95,4 |
| Number of young girls (15- 19 years old) surveyed | 3312 | 1891 | 1830 |
| Number of girls (20-24 years old) surveyed | 3311 | 1845 | 1992 |
| Number of young girls (15- 24 years old) surveyed | 6623 | 3736 | 3822 |
